# Transcranial direct current stimulation over the posterior parietal cortex improves visuomotor performance and proprioception in the lower extremities

**DOI:** 10.3389/fnhum.2022.876083

**Published:** 2022-08-18

**Authors:** Yasushi Kamii, Sho Kojima, Hideaki Onishi

**Affiliations:** ^1^Graduate School, Niigata University of Health and Welfare, Niigata, Japan; ^2^Institute for Human Movement and Medical Sciences, Niigata University of Health and Welfare, Niigata, Japan; ^3^Department of Physical Therapy, Niigata University of Health and Welfare, Niigata, Japan

**Keywords:** transcranial direct current stimulation, posterior parietal cortex, neuromodulation, motor control, proprioception

## Abstract

The purpose of this study was to examine whether anodal transcranial direct current stimulation (a-tDCS) over the posterior parietal cortex (PPC) could affect visuomotor performance and proprioception in the lower extremities. We evaluated visuomotor performance in 15 healthy volunteers using a visuomotor control task by plantar dorsiflexion of the ankle joint, and calculated the absolute difference between the target and measured angle. In addition, we evaluated proprioception using a joint position matching task. During the task, the subject reproduced the ankle joint plantar dorsiflexion angle presented by the examiner. We calculated the absolute difference between the presented and measured angles (absolute error) and the variation of measured angles (variable error). Simultaneously, a-tDCS (1.5 mA, 15 min) or sham stimulation was applied to the right PPC. We observed that the absolute error of the visuomotor control task and the variable error of the joint position matching task significantly decreased after a-tDCS. However, the absolute error of the joint position matching task was not affected. This study suggests that a-tDCS over the PPC improves visuomotor performance and reduces the variable error in the joint position matching task.

## Introduction

Recently, transcranial direct current stimulation (tDCS) has become increasingly recognized as an external stimulation method for non-invasive brain activity modulation. Anodal stimulation is supposed to increase and cathodal stimulation to decrease the excitability of the stimulated brain region (Nitsche and Paulus, 2000; Liebetanz et al., 2002; Nitsche et al., 2003a,b; Ardolino et al., 2005). a-tDCS over the primary motor cortex (M1) reportedly improved visuomotor control task performance using the one hand (Kwon et al., 2015), and over the supplementary motor area (SMA), it enhances visuomotor control task learning using the one hand (Vollmann et al., 2013). However, Karabanov et al. (2021) reported that tDCS over the M1 does not affect visuomotor control task. In addition, a-tDCS over the primary somatosensory (S1) also improves joint positional sense function in the upper limb (Muffel et al., 2019) and lowers foot sole vibratory thresholds (Zhou et al., 2018). In contrast, there was a considerable variance in the effect of tDCS over S1 on joint position matching task (Muffel et al., 2019).

In this context, previous research showed that the posterior parietal cortex (PPC) might be involved in motor control in visuomotor control tasks and sensory control including the joint position sense. The PPC is located between the S1 and the visual cortices (Culham et al., 2006), and is reportedly connected to premotor and visual cortices, S1, and other brain regions (Whitlock, 2017). In addition, PPC activity has been reported to depend on the motor task (Sack et al., 2002; Koeneke et al., 2004; Iandolo et al., 2018; Fitzpatrick et al., 2019; Karabanov et al., 2021). In previous functional magnetic resonance imaging (fMRI)-based studies, a significant increase in activity in the right superior parietal lobule (SPL) (Koeneke et al., 2004; Karabanov et al., 2021) and right inferior parietal lobule (Koeneke et al., 2004), both part of the PPC, could be observed during a task in which subjects had to adjust their left finger muscle output to a visual target. In addition, activity in the right SPL reportedly increased when subjects performed a task in which they reproduced an ankle joint angle presented in advance, compared to a task in which subjects performed repetitive dorsiflexion of the ankle joint while maintaining the pace at 1 Hz using auditory feedback (Iandolo et al., 2018). Furthermore, a significant correlation between activity in the right PPC and joint position matching task performance using the left ankle has been reported (Iandolo et al., 2018). Therefore, PPC activity is thought to be involved in motor coordination tasks matching visual targets or requiring joint position sense. Furthermore, a-tDCS over the PPC improved postural control function (Young et al., 2020) and visual processing (Zhu et al., 2021), which are thought to be related to PPC activity. These results suggest that a-tDCS over PPC might improve visuomotor control task performance and joint positional function, which are known to be involved in both visuomotor control and joint positional sense functions. In addition, numerous studies have reported on the effects of tDCS on visuomotor control tasks and joint position sense in the upper extremities (Friel et al., 2017; Cole et al., 2018). In patients with neurological diseases, joint position sense of the lower extremity is associated with the rate of falls (Guillochon et al., 2010), and a decline in motor control of the lower extremities is associated with a decline in walking ability (Tanaka et al., 2009). Therefore, the development of stimulation methods that improve joint position sense and visuomotor control of the lower extremity has potential clinical significance.

Therefore, we hypothesized that a-tDCS over the PPC would increase PPC excitability, thereby improving visuomotor control task performance and joint position sense function. Therefore, this study aimed to clarify how a-tDCS over the PPC could affect visuomotor control task performance and joint position sense in the lower extremities.

## Materials and methods

### Subjects

Overall, 15 healthy volunteers [aged 21–24 years; mean ± standard deviation (SD): 22.2 ± 0.9 years; 14 men and 1 woman] participated in this study. We determined the sample size by referring previous studies that were used as part of the crossover study with healthy adults to examine the effect of tDCS (Ohn et al., 2008; Binkofski et al., 2011; Wardzinski et al., 2019). Inclusion criteria were no previous history of any ankle-related orthopedic impairment. This study was conducted after orally explaining the contents of the study to the subjects and obtaining their informed consent. In addition, this study was approved by the Ethics Committee of Niigata University of Health and Welfare and was conducted in accordance with the Declaration of Helsinki.

### Limb measurement and experimental protocol

**FIGURE 1:**
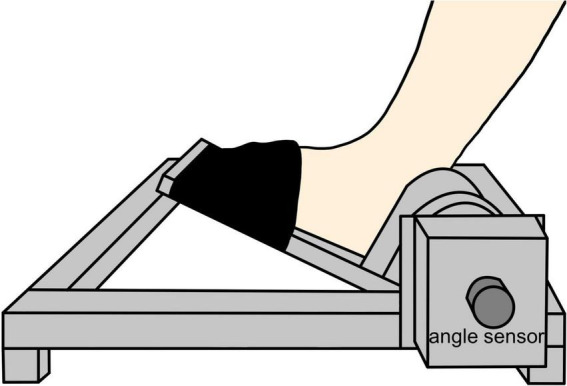
Foot and equipment fixation. The distal part of the left foot from the metatarsal bone was fixed to the ankle arthrometer with a belt at 10° of ankle joint plantar flexion.

**FIGURE 2:**
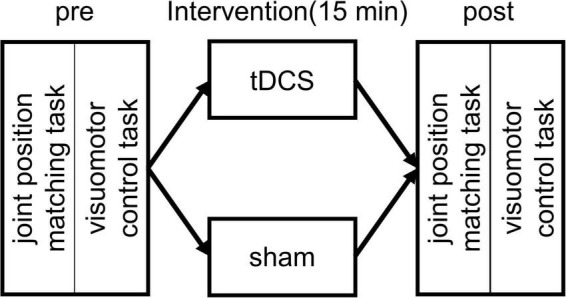
Experimental protocol. At the beginning of each experiment, the subjects performed an ankle joint position matching and a visuomotor control task. This was followed by a 15 min intervention with transcranial direct current stimulation (tDCS) or sham stimulation. After the intervention, the subjects performed again the joint position matching and the visuomotor control task.

The measurement position was a resting sitting position with a knee joint flexion and ankle joint plantar flexion of 80° and 10°, respectively. The waist and left foot were fixed to a seat and a footplate (S-19103; Takei Scientific Instruments, Niigata, Japan) (Figure 1), respectively, to maintain the posture of the subjects during the experiment. The left foot was fixed with a belt distal to the metatarsal bone. As for the experimental procedure, we performed first the joint position matching task of the ankle joint and the visuomotor control task. This was followed by a 15-min intervention. After the intervention, the subjects performed again the joint position matching and the visuomotor control tasks (Figure 2). This study had a crossover design, and we randomly applied two types of intervention to the same subject on separate days.

### Joint position matching task

**FIGURE 3:**
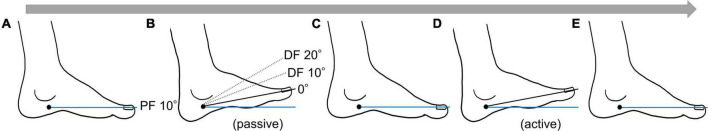
One trial flow of the joint position matching task. **(A)** Starting position (ankle joint plantar flexion of 10°). **(B)** Target angle presentation. The examiner presented the target angle by moving the ankle joint of the subject. **(C)** The examiner returned the subject’s ankle joint to the starting position. **(D)** The subject voluntarily dorsiflexed the ankle joint to the presented target angle. **(E)** At the examiner’s signal, the subject returned the ankle joint to the starting position.

To evaluate the joint position sense, we used a joint position matching task in which the subject reproduced the plantar dorsiflexion angle of the ankle joint presented by the examiner using voluntary movements. During the task, the joint angle signals were recorded on a personal computer with a sampling frequency of 4 kHz by an A/D converter (PowerLab 8/30; AD Instruments, CO, United States). We applied three presentation angles: 0°, 10°, and 20° dorsiflexion (DF) of the ankle joint. The measurement procedure was as follows: first, from the starting limb position of 10° plantar flexion (Figure 3A), the examiner moved the left ankle joint of the subject to one of the three presented angles (Figure 3B), presented the angle for 3 s, and then returned to the original starting limb position (Figure 3C). In this study, the examiner manually moved the footplate to which the subject’s foot was fixed to provide the subject with a target angle. The footplate had a stopper to limit the dorsiflexion angle; the maximum dorsiflexion angle could be set by positioning the stopper. The examiner changed the stopper position for each target angle and moved the footplate to the position where it met the stopper to accurately mark the target angle. The subject then voluntarily dorsiflexed the left ankle joint to the same angle as the angle presented by the examiner and held the position that he felt was the same as the presented angle for 3 s (Figure 3D). After that, the subject returned the ankle joint to the original starting limb position following the signal of the examiner (Figure 3E). This series of movements was defined as one trial, and five trials were conducted in the case of each angle, for a total of 15 trials in random order.

### Visuomotor control task

**FIGURE 4:**
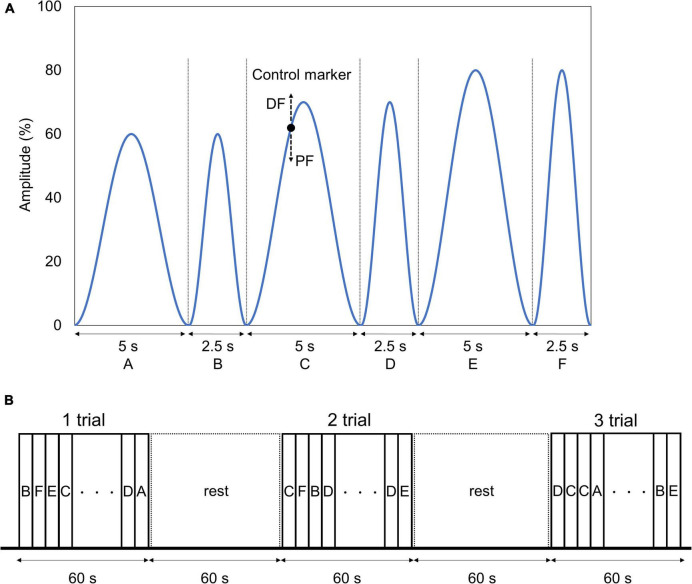
Visuomotor control task. **(A)** Target waveform and control marker. The blue line and the black point indicate the six target waveforms used in the task and the marker controlled by the subject, respectively. The target waveform was presented while moving from right to left on the monitor. The marker was set to move up and down according to the dorsal flexion and plantar flexion of angle of the subject. The subject was instructed to adjust the ankle plantar dorsiflexion angle so that the marker overlapped the target waveform as much as possible. **(B)** Visuomotor control task order. One trial of the task was set to 60 s, and three trials were conducted before and after the intervention. We ensured a 60-s resting period between each trial. The waveforms were presented in random order in one trial, with waveforms A, C, and E being presented twice each, and waveforms B, D, and F being presented four times each.

We applied a visuomotor control task by dorsiflexion of the left ankle joint to evaluate the lower extremity motor functions. For the task, we used a waveform control software (S-17226; Takei Scientific Instruments, Niigata, Japan) and an A/D converter (TSA-210; Takei Scientific Instruments, Niigata, Japan) to record the ankle joint angles on a personal computer at a sampling frequency of 100 Hz. The starting limb position was 80° flexion and 10° plantar flexion of the knee and the ankle joint, respectively. The subject performed plantar dorsiflexion movements of the left ankle joint so that the marker, which moved up and down according to the plantar dorsiflexion angle of the ankle joint, overlapped as accurately as possible with the target waveform presented on a monitor set up in front of the subject. The presented waveforms were based on the starting limb position of 10° plantar flexion of the ankle joint, and the dorsiflexion position of 20° was defined as 100%, consisting of six patterns: A (0–60% for 5 s), B (0–60% for 2.5 s), C (0–70% for 5 s), D (0–70% for 2.5 s), E (0–80% for 5 s), and F (0–80% for 2.5 s) (Figure 4A). One task trial was set to 60 s, and three trials were conducted before and after the intervention. A rest period of 60 s was ensured between each trial. The waveforms of patterns A, C, and E were presented twice each, and the waveforms of B, D, and F were presented four times each randomly in one trial (Figure 4B). We created the waveform pattern by referring to previous studies that conducted visuomotor control tasks using fingers (Ishikawa et al., 2018; Abe et al., 2019; Miyaguchi et al., 2019) and the ankle joint (Sriraman et al., 2014).

### Transcranial direct current stimulation

Transcranial direct current stimulation was delivered by a direct current stimulator (Eldith; neuroConn GmbH, Ilmenau, Germany) through a pair of saline-soaked sponge electrodes (5 cm × 5 cm, 25 cm^2^). The anodal and cathodal electrodes were placed in the right PPC and on the left orbit, respectively. The position of the right PPC was set as the position of P4 determined with the International system 10–20 with reference to a previous study (Lo et al., 2019). The stimulus intensity was set at 1.5 mA, and the duration of current application was set to 15 min (Li et al., 2017), with fade in and fade out times of 15 s. Two conditions were established for the tDCS: one in which we delivered a 15-min stimulation (tDCS condition) and another with stimulation of 30 s (sham condition). Each intervention was randomly administered to the same subject at intervals of at least 1 week to avoid carryover effect with reference to previous studies (Minarik et al., 2015; Sugawara et al., 2015; Alix-Fages et al., 2021; Farnad et al., 2021).

### Data analysis

For the joint position matching task, we calculated absolute and variable errors as per Schmidt and Lee (2011). We calculated the absolute error by first converting into absolute values the difference between the presented angle and the angle reproduced by the subject and then calculating the absolute error for each trial. For the angle reproduced by the subject, we calculated the average value of the angle for 3 s when the subject reproduced the angle for each trial and used that value as the angle reproduced by the subject. Then, we averaged the absolute errors of the 15 trials before and after the intervention to calculate the pre- and post-intervention absolute errors, respectively. We calculated the variable error by dividing the angle actually reproduced by the subject before and after the intervention by each of the three presentation angles and calculating the standard deviation of the angle for five trials each. Finally, we averaged the standard deviations for each of the three presentation angles before and after the intervention to calculate the pre- and post-intervention variable errors, respectively.

For the visuomotor control task, we calculated the absolute and variable errors. We first converted the results of the visuomotor control task into absolute values of the difference between the target and the measured angles, and then calculated the absolute error for each trial. We then determined the mean values of the absolute errors of the three trials before and after the intervention to calculate the pre- and post-intervention absolute errors, respectively. We calculated the variable error before and after the intervention by using the standard deviation of the difference between the target and the measured angles for each trial. The target angles of visuomotor control task change by the minute, so the measured angles also vary widely. Therefore, we used the difference between the target and the measured angles to calculate the variable error in place of measured angles. Finally, we averaged the standard deviations for three trials before and after the intervention to calculate the pre- and post-intervention variable errors, respectively.

### Statistical analysis

We performed the statistical analyses using SPSS statistics Ver. 27 (IBM SPSS, Armonk, NY, United States). The Shapiro–Wilk test revealed that the absolute error, and the variable error in both tasks did not follow normality. In numerous previous studies involved crossover designs, and their data did not follow normal distribution, they performed the Wilcoxon signed-rank test (Suarez et al., 2001; Tanen et al., 2008; Hefner et al., 2016; Shah et al., 2021). Therefore, we also performed the Wilcoxon signed-rank test between Pre and Post of each intervention condition with reference to these studies. In addition, we analyzed the absolute error of visuomotor control task and variable error of joint position matching task using a generalized linear mixed model (GLMM) for the main effects of time (before or after intervention) and stimulation condition (tDCS or sham condition), and interaction effect (time × stimulation condition) with participant as a random effect. The statistical significance was set at a *P*-value of <0.05.

## Results

### Visuomotor control task

**FIGURE 5:**
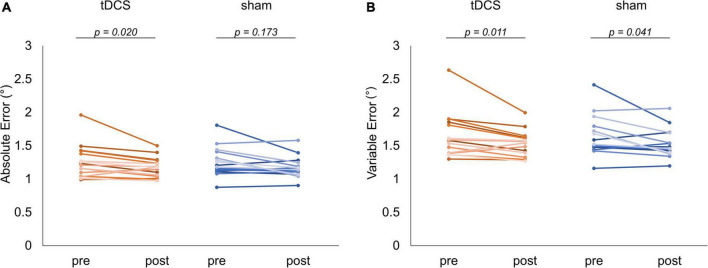
Comparison of visuomotor control task performance. The red and blue lines indicate the performance of each subject in the task for each condition, respectively. **(A)** Absolute error comparison. In the case of the transcranial direct current stimulation (tDCS) condition, a significant decrease could be detected in the variable error after the intervention (*p* = 0.020). **(B)** Variable error comparison. The significantly decrements observed in the case of the tDCS (*p* = 0.011) and sham (*p* = 0.041) conditions.

Figure 5 shows the absolute and variable error changes before and after each intervention condition. In the case of the tDCS condition, the absolute error mean values (mean ± SD) were 1.26 ± 0.25° (Pre) and 1.18 ± 0.15° (Post). In the case of the sham condition, the values were 1.25 ± 0.22° (Pre) and 1.17 ± 0.16° (Post). The Wilcoxon signed-rank test revealed that the post-intervention absolute error significantly decreased compared to that of the pre-intervention in the case of the tDCS condition (*p* = 0.020, *r* = −0.601). In contrast, we observed no significant difference in the case of the sham condition before and after the intervention (*p* = 0.173, *r* = −0.352) (Figure 5A). Meanwhile, in the case of the tDCS condition, the variable error mean values were 1.65 ± 0.33° (Pre) and 1.52 ± 0.20° (Post). In the case of the sham condition, the values were 1.64 ± 0.29° (Pre) and 1.53 ± 0.20° (Post). The Wilcoxon signed-rank test revealed that the post-intervention variable error significantly decreased compared to that of the pre-intervention in the case of the tDCS (*p* = 0.011, *r* = −0.66) and sham (*p* = 0.041, *r* = −0.53) conditions (Figure 5B). Additional results related to the absolute error of each waveform pattern, the statistical analyses included the GLMM for carryover and sequence effects can be found in the Supplementary Material (Supplementary Tables 1–4).

### Joint position matching task

**FIGURE 6:**
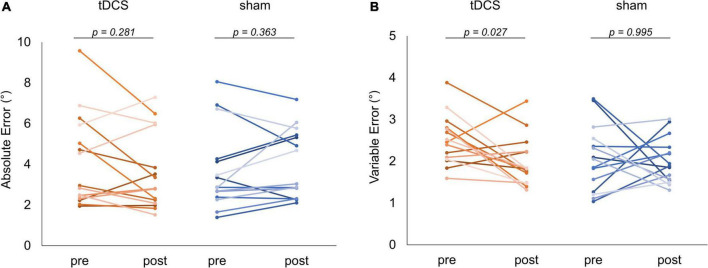
Comparison of joint position matching task performance. The red and blue lines indicate the performance of each subject in the task for each condition, respectively. **(A)** Absolute error comparison. No significant change was observed in the case of any condition. **(B)** Variable error comparison. In the case of the transcranial direct current stimulation (tDCS) condition, a significant decrease could be detected in the variable error after the intervention (*p* = 0.027).

Figure 6 shows the absolute and variable error changes before and after each intervention condition. In the case of the tDCS condition, the absolute error mean values were 4.15 ± 2.25° (Pre) and 3.60 ± 1.90° (Post). In the case of the sham condition, the values were 3.63 ± 1.96° (Pre) and 3.91 ± 1.65° (Post). The Wilcoxon signed-rank test revealed no significant difference in the case of the tDCS (*p* = 0.281, *r* = −0.279) and sham (*p* = 0.363, *r* = 0.235) conditions before and after the intervention (Figure 6A). Meanwhile, in the case of the tDCS condition, the variable error mean values were 2.50 ± 0.59° (Pre) and 1.98 ± 0.58° (Post). In the case of the sham condition, the values were 2.05 ± 0.78° (Pre) and 1.99 ± 0.54° (Post). The Wilcoxon signed-rank test revealed that the post-intervention variable error significantly decreased compared to that of the pre-intervention in the case of the tDCS condition (*p* = 0.027, *r* = −0.572). In contrast, no significant difference was observed in the case of the sham condition before and after the intervention (*p* = 0.995, *r* = −0.015) (Figure 6B). Additional results related to the statistical analyses included the GLMM for carryover and sequence effects, and the absolute error and variable error of each stimulation condition can be found in the Supplementary Material (Supplementary Tables 2–5).

## Discussion

We investigated whether tDCS over the PPC would affect visuomotor control task performance and joint position sense. The tDCS reduced the absolute error of the visual control task post-intervention compared with pre-intervention, whereas the variable error of visuomotor task decreased significantly under both conditions. Moreover, the tDCS reduced the variable error of the joint position matching task post-intervention compared with pre-intervention. However, no similar significant change was observed in the case of the sham condition. In addition, the absolute error of the joint position matching task did not change significantly under any conditions.

### Transcranial direct current stimulation effect on the visuomotor control task

The visuomotor control task absolute error significantly decreased post-intervention in the case of the tDCS condition compared to pre-intervention, indicating that the visuomotor control task performance improved in the case of the tDCS condition. In previous studies, M1, SMA, and PPC were significantly activated during visuomotor tasks (Koeneke et al., 2004; Karabanov et al., 2021). Moreover, tDCS over M1 improved visuomotor control task performance (Kwon et al., 2015), and over SMA, it enhanced visuomotor control task learning (Vollmann et al., 2013). Therefore, increasing the excitability of brain regions involved in visuomotor control can improve visuomotor control performance. Furthermore, the PPC, used for stimulation in this experiment, was involved in motor control (Joodaki et al., 2001) based on visual information (Tunik et al., 2005; Buneo and Andersen, 2006). Therefore, we considered that the anodal tDCS over the PPC in this study increased PPC excitability, which in turn improved the visuomotor control task performance.

However, the variable error of the visuomotor control task significantly decreased post-intervention in both cases of the tDCS and sham stimulation conditions. This decrease could be attributable to the fact that motor learning occurred through the six trials performed before and after intervention. A previous study reported that more feedback is better than less for motor learning (Fujii et al., 2016). Throughout the visuomotor task, subjects receive the feedback of the waveform and position of the control maker in this study. It is possible that they get better with each trial, and the difference between the target and the measured angles converge. Therefore, it is thought that the motor learning causes the decrease in the variable error of visuomotor control task under both conditions.

### Transcranial direct current stimulation effect on the joint position matching task

The variable error of the joint position matching task significantly decreased post-intervention in the case of the tDCS condition compared to pre-intervention. The variable error is supposed to reflect response consistency within the subject (Schmidt and Lee, 2011). Therefore, the results of this study indicate that in the case of the tDCS condition, the responses of each subject were more consistent after than before the intervention. Previous studies reported that the activity of the right SPL, which is part of the PPC, increased during a joint position matching task, and that a significant negative correlation could be observed between the activity of the right SPL during a joint position matching task using the left lower limb and the variable errors obtained in that task (Iandolo et al., 2018). The results showed that the variable error is smaller for subjects with higher SPL activity during the joint position matching task. Therefore, our study suggests that the anodal tDCS on the right PPC might have increased the excitability of the right SPL, which might have reduced the variable error of the joint position matching task after the intervention.

However, the absolute error of the joint position matching task did not change significantly in the case of either the tDCS or sham stimulation conditions. The subject’s age, the task difficulty, and individual differences in stimulating effects are probably related to this result. First, our measurements were performed in healthy young adults, aged 22.2 ± 0.9 years. A previous study that compared absolute errors in a joint position matching task by age reported that the absolute errors of the 20–30-year-old group were smaller than those of other age groups (Goble, 2010). In addition, a previous study comparing ankle joint position matching task learning between young adults and the elderly showed that learning occurred in the elderly, but not in young adults (Madhavan and Shields, 2005). Furthermore, the average absolute error before stimulation for each stimulation condition in this study was smaller than that of previous studies (Iandolo et al., 2018; Ikarashi et al., 2020) that measured joint position matching task similarly to the present study. These results suggest that the absolute error might not have changed in this study on young adults as the joint position sense function of the subjects was high from the beginning. Second, we also used a joint position matching task in which the angle presented to one ankle joint was reproduced by the ipsilateral ankle joint in this study. Previous studies comparing the absolute errors of different joint position matching task forms have reported that the degree of difficulty varies depending on the joint position matching task form (Goble, 2010; Iandolo et al., 2018). In particular, a previous study using the ankle joint reported that the absolute error in the angle reproducing task presented to the ipsilateral ankle joint is smaller and less difficult than that presented to the contralateral ankle joint (Iandolo et al., 2018). Therefore, it is possible that in this change could be observed due to the ease of the task. Third, we used tDCS for stimulation of the PPC. Previous studies on tDCS have reported individual differences in its effects (López-Alonso et al., 2014; Wiethoff et al., 2014). Therefore, the lack of changes to the absolute error in this study might be due to individual differences.

### Clinical site significance

In this study, the tDCS over the PPC reduced the absolute error in the visuomotor control task and the variable error in the joint position matching task. Previous studies on patients with stroke reported impaired visuomotor control function (Lodha et al., 2010; Chang et al., 2013) and joint position sense (Connell et al., 2008; Yang and Kim, 2015) after stroke. To date, tDCS over the M1 has been reported to improve visuomotor control function (Kwon et al., 2015) and to improve joint position sense when applied over the S1 (Muffel et al., 2019). To the best of our knowledge, this is the first study to simultaneously examine how tDCS over the PPC affects visuomotor control task and joint position matching task performance. Therefore, the intervention used in this study is a new method that simultaneously approaches motor and sensory functions, suggesting it might be effective in improving both visuomotor control and joint position sense. However, this study involved healthy adult subjects, and it is unclear whether it can be adapted to patients with impaired visuomotor control and joint position sense. Therefore, the effect of the intervention on patients with impaired motor function and joint position sense requires further investigation.

### Study limitations

This study has several limitations. First, the cortical activities of PPC, S1, M1, and prefrontal cortex were not measured, and the position of the electrodes of this study may have resulted in the changing of electric field in these regions that exist between the electrodes. Therefore, it is conceivable that brain activity measurements would be required in future studies to confirm the actual PPC and other regions activity modulation. Second, we targeted only the PPC, and there was insufficient consideration of changes in other brain regions. In the future, comparison between the effects achieved when tDCS is applied to the PPC, other regions, and will help determine more effective stimulation parameters. Third, the subjects’ foot dominance was not investigated, and the relationship between the stimulation effect and lower extremity dominance needs to be unveiled in the future. Fourth, the sex ratio of the subjects in this study was biased and this may have affected our results. Previous studies have showed differing results of electric field simulation (Kuo et al., 2006; Bhattacharjee et al., 2022) and the effect of tDCS (Thomas et al., 2019) in men and women. To further generalize the results of this study, it is necessary to confirm the results of this study in a bigger sample of female subjects. Finally, it is still debatable on the carryover and the sequence effects. Our Supplementary Data showed that there was no significant intervention effect (time × stimulation condition) and there was no difference in the pre-intervention values of second experimental phase regardless of whether subjects received the a-tDCS or the sham stimulation in the first experimental phase (Supplementary Tables 2, 3). Moreover, tDCS could reportedly lead to subsequent behavioral and cortical changes lasting for 90 min (Nitsche and Paulus, 2000, 2001), and each intervention was randomly administered to the same subject at intervals of at least 1 week in this study. Owing to these reasons, we consider that there are little to affect the results of this study. On the other hand, another Supplementary Data showed that there was sequence effect of absolute error of visuomotor control task (Supplementary Table 4). However, we randomly applied two types of intervention to the same subject, and there was a relatively even number of those who received a-tDCS or those who received sham stimulation in the first experimental phase. Under the situation, the post-intervention absolute error of visuomotor control task significantly decreased compared to that of the pre-intervention in the case of only the tDCS condition. Therefore, we consider that the tDCS affected our result greater than sequence effect.

## Conclusion

We investigated how tDCS over the PPC affects visuomotor control task performance and joint position sense. Our results suggest that a-tDCS over PPC improves visuomotor control task performance and reduces variable errors in joint position matching task.

## Data availability statement

The raw data supporting the conclusions of this article will be made available by the authors, without undue reservation.

## Ethics statement

The studies involving human participants were reviewed and approved by the Ethics Committee of Niigata University of Health and Welfare. The patients/participants provided their written informed consent to participate in this study.

## Author contributions

YK and SK designed the experiment, recorded and analyzed the data, and wrote the manuscript. HO designed the experiment and edited and revised the manuscript. All authors contributed to the article and approved the submitted version.

## Abbreviations

PPC: posterior parietal cortex
SMA: supplementary motor area
M1: primary motor cortex
S1: primary somatosensory cortex
SPL: superior parietal lobule
IPL: inferior parietal lobule
tDCS: transcranial direct current stimulation
fMRI: functional magnetic resonance imaging
GLMM: generalized linear mixed model.
