# Supplementary material for: Transcranial direct current stimulation over the posterior parietal cortex improves visuomotor performance and proprioception in the lower extremities
Source: Front Hum Neurosci. 2022 Aug 18;16:876083. doi: 10.3389/fnhum.2022.876083 (PMC9434688; doi:10.3389/fnhum.2022.876083)
Supplement: Supplementary file 1 [file Data_Sheet_1.docx]

Supplementary Material

**Supplementary table 1.** Absolute error of each waveform pattern for each stimulation condition in the visuomotor control task.

|  |  | **pre** | **post** | ***p*** | ***r*** |
| --- | --- | --- | --- | --- | --- |
| **tDCS**  **condition** | pattern A | 0.96 ± 0.21° | 0.89 ± 0.12° | 0.363 | -0.235 |
|  | pattern B | 1.34 ± 0.25° | 1.24 ± 0.21° | 0.047* | -0.513 |
|  | pattern C | 1.08 ± 0.18° | 1.00 ± 0.19° | 0.009** | -0.675 |
|  | pattern D | 1.42 ± 0.36° | 1.33 ± 0.19° | 0.156 | -0.367 |
|  | pattern E | 1.22 ± 0.28° | 1.11 ± 0.20° | 0.88 | -0.44 |
|  | pattern F | 1.55 ± 0.33° | 1.48 ± 0.24° | 0.307 | -0.264 |
| **sham**  **condition** | pattern A | 0.95 ± 0.24° | 0.85 ± 0.12° | 0.112 | -0.411 |
|  | pattern B | 1.27 ± 0.25° | 1.21 ± 0.23° | 0.609 | -0.132 |
|  | pattern C | 1.05 ± 0.30° | 0.99 ± 0.24° | 0.233 | -0.308 |
|  | pattern D | 1.36 ± 0.24° | 1.38 ± 0.23° | 0.733 | 0.088 |
|  | pattern E | 1.27 ± 0.30° | 1.08 ± 0.19° | 0.001** | -0.821 |
|  | pattern F | 1.60 ± 0.22° | 1.50 ± 0.21° | 0.156 | -0.367 |

*p < 0.05, **p < 0.01

The Shapiro–Wilk test revealed that the absolute error of each waveform pattern did not follow normality. Therefore, we performed the Wilcoxon signed-rank test between Pre and Post of each waveform pattern. The Wilcoxon signed-rank test revealed that the post-intervention absolute error significantly decreased compared to that of the pre-intervention in the pattern B (*p*= 0.047 *r* = −0.513), pattern C (*p*= 0.009 *r* = −0.675) in tDCS condition. On the other hand, the post-intervention absolute error significantly decreased compared to that of the pre-intervention in pattern E (*p*= 0.001 *r* = −0.821).

**Supplementary table 2.** Absolute error of visuomotor control task and variable error of joint position matching task for each stimulation condition.

|  | **stimulation condition** | **pre** | **post** |
| --- | --- | --- | --- |
| **absolute error of visuomotor control task** | tDCS condition | 1.26 ± 0.25° | 1.18 ± 0.15° |
|  | sham condition | 1.25 ± 0.22° | 1.17 ± 0.16° |
| **variable error of joint position matching task** | tDCS condition | 2.50 ± 0.59° | 1.98 ± 0.58° |
|  | sham condition | 2.05 ± 0.78° | 1.99 ± 0.54° |

To clarify the carryover effect, we analyzed the absolute error of visuomotor control task and variable error of joint position matching task using a generalized linear mixed model (GLMM) for the main effects of time (before or after intervention) and stimulation condition (tDCS or sham condition), and interaction effect (time ×stimulation condition) with participant as a random effect. As a result, there was significant main effect of time (*F*(1, 42) = 5.693, *p* = 0.022). However, no significant main effect of stimulation condition (*F*(1, 42) = 0.059, *p* = 0.809) and interaction effect (*F*(1, 42) = 0.002, *p* = 0.965) in absolute error of visuomotor control task were observed. In addition, no significant main effects of time (*F*(1, 42) = 3.925, *p* = 0.054), stimulation condition (*F*(1, 42) = 2.169, *p* = 0.148), and interaction effect (*F*(1, 42) = 2.415, *p* = 0.128) in variable error of joint position matching task could be observed.

**Supplementary table 3.** Pre-intervention absolute error of visuomotor control task and pre-intervention variable error of joint position matching task, in the second experimental phase.

|  | **received the anodal tDCS in the first experiment** | **received the sham stimulation in the first experiment** | ***p*** | ***r*** |
| --- | --- | --- | --- | --- |
| **absolute error of visuomotor control task** | 1.20 ± 0.25° | 1.10 ± 0.07° | 0.463 | -0.209 |
| **variable error of joint position matching task** | 2.25 ± 0.84° | 2.27 ± 0.36° | 0.950 | -0.020 |

To clarify the carryover effect clearly, we compared the pre-intervention absolute error of visuomotor control task and variable error of joint position matching task, in the second experimental phase between the subjects who received the anodal tDCS in the first experimental phase, and those who received the sham stimulation in the first experimental phase. We analyzed the pre-intervention absolute error of visuomotor control task using the Wilcoxon rank sum test. We analyzed the pre-intervention variable error of joint position matching task using the Independent-samples t-test. As result, there was no significant differences in the pre-intervention absolute error of visuomotor control task (*p* = 0.463, *r* = −0.209), and pre-intervention variable error of joint position matching task (*p* = 0.950, *r* = 0.020).

**Supplementary table 4.** Absolute error of visuomotor control task and variable error of joint position matching task for each experimental phase.

|  | **First phase** | | **Second phase** | |
| --- | --- | --- | --- | --- |
|  | **pre (set 1)** | **post (set 2)** | **pre (set 3)** | **post (set 4)** |
| **absolute error of visuomotor control task** | 1.36 ± 0.22° | 1.23 ± 0.16° | 1.15 ± 0.19° | 1.12 ± 0.12° |
| **variable error of joint position matching task** | 2.30 ± 0.75° | 1.99 ± 0.52° | 2.26 ± 0.66° | 1.97 ± 0.56° |

To clarify the sequence effect clearly, we analyzed the absolute error of visuomotor control task and variable error of joint position matching task using the Friedman test for through the whole phase. Post hoc analyses were performed using the Bonferroni correction. The Friedman test showed that there was significant difference in absolute error of visuomotor control task (*x²*= 29.160, *p* < 0.001, *n* =15). Post hoc analyses revealed there were significant difference between pre-intervention in first phase and pre-intervention in second phase (*p* = 0.001), pre-intervention in first phase and post-intervention in second phase (*p* < 0.001), post-intervention in first phase and post-intervention in second phase (*p* = 0.007). However, no significant difference in variable error of joint position matching task could be observed (*x²*= 3.320, *p* = 0.345, *n* =15).

**Supplementary table 5.** Absolute error and variable error for each stimulation condition in the joint position matching task.

|  |  | **pre** | **post** | ***p*** | ***r*** |
| --- | --- | --- | --- | --- | --- |
| **tDCS**  **condition** | absolute error | 4.15 ± 2.25° | 3.60 ± 1.90° | 0.281 | -0.279 |
|  | variable error | 2.50 ± 0.59° | 1.98 ± 0.58° | 0.027* | -0.572 |
| **sham**  **condition** | absolute error | 3.63 ± 1.96° | 3.91 ± 1.65° | 0.363 | -0.235 |
|  | variable error | 2.05 ± 0.78° | 1.99 ± 0.54° | 0.995 | -0.015 |

*p < 0.05
